# Influence of mothers’ and frontline health workers’ knowledge, attitude, and practices on infant and young child feeding and child nutrition: a cross-sectional study in aspirational districts of Assam, India

**DOI:** 10.3389/fnut.2024.1413867

**Published:** 2024-12-16

**Authors:** Sonali Randhawa, Manisha Choudhury, Devika Gali Choudhary, Ramnath Ballala, Shailendra Hegde, Priyanka Barman, Vishal Dogra

**Affiliations:** ^1^Health Systems Transformation Platform, New Delhi, India; ^2^Department of Food Science and Nutrition, College of Community Science, Assam Agricultural University, Jorhat, India; ^3^Piramal Foundation, Hyderabad, India; ^4^Bharatiya Vikas Trust, Manipal, India; ^5^Piramal Swasthya Management and Research Institute, Hyderabad, India; ^6^National Institute of Food Technology, Entrepreneurship and Management, Thanjavur (NIFTEM), Guwahati, India; ^7^Bill & Melinda Gates Foundation, New Delhi, India

**Keywords:** IYCF practices, knowledge, attitude, practices, undernutrition, aspirational districts, Assam

## Abstract

The knowledge, attitude, and practices (KAP) of mothers and frontline health workers (FLWs) regarding optimal Infant and Young Child Feeding (IYCF) are essential for mitigating undernutrition and associated morbidities among under-five children. The study assessed the KAP of mothers (of children aged 0–60 months) and FLWs regarding recommended IYCF practices, the association of mother’s KAP with their demographic characteristics and children’s nutritional outcomes, and the prevalence of stunting, wasting, and underweight among children aged 0–60 months in five high-focus districts of Assam. Data were collected from 389 mothers, 456 children, and 138 FLWs using a standard method. Of the 389 mothers, 29% had good KAP scores, 42% scored average, and 29% had poor KAP scores regarding IYCF practices. The mean KAP score increased significantly with improvements in variables such as community, language, education level, monthly income, and living conditions (*p* < 0.01). Less than half of the children were stunted (40%), one-third were affected by wasting (28%), and approximately 43% were underweight. Children whose mothers had a KAP score of less than 40% were 2.05 times more likely to experience stunting (CI = 1.04–4.02) than those whose mothers scored above 60%. Similar findings were reported for wasting and underweight. Of the 138 FLWs, 56% had good KAP scores, 30% had average scores, and 14% had poor scores. There was a noticeable gap in the KAP of the mothers regarding IYCF practices compared to that of FLWs. A comprehensive intervention plan to improve feeding practices can enhance the nutritional status of under-five children.

## 1 Introduction

Malnutrition is a critical public health concern influencing child morbidity and mortality, accounting for one-third (35%) of global deaths and two-thirds (68%) of deaths among under-five children in India (1). Globally, 25% (approximately 159 million) of under-five children are stunted, 16% are underweight (101 million), and 11% are wasted (52 million). South Asia has 38% of children who are stunted and 33% who are wasted, with India contributing significantly: 32.1% of children are underweight and 35.5% are stunted, largely due to chronic undernutrition (2–4).

Although India’s infant mortality rate has decreased from 89 per 1,000 live births (in 1990) to 28 (in 2020) per 1,000 live births, progress in managing undernutrition remains unsatisfactory. For instance, the rate of wasting changed only marginally from 19.8% (NFHS 2005–2006) to 19.3% (NFHS 2019–21) in the last decade (2, 5, 6). In Assam, a northeastern Indian state, the indicators are even more concerning: 35.3% of under-five children are stunted, 32.8% are underweight, 21.7% are wasted, and 9.1% are severely wasted, reflecting a decline compared to the 2015–16 NFHS data (2, 7, 8). Key nutrition indicators for India, Assam, and globally are provided in Table 1.

**Table 1:** Key nutrition indicators globally, in India, and in Assam.

Indicators (in %)	Globally	India		Assam	
	2019	NFHS-IV (2015–16)	NFHS-V (2019–21)	NFHS-IV (2015–16)	NFHS-V (2019–21)
Wasting	6.9	21	19.3	17	21.7
Underweight	13	35.7	32.1	29.8	32.8
Stunting	21.3	38.4	35.5	36.4	35.3

Sources: The World Bank (3); International Institute for Population Sciences (2); International Institute for Population Sciences (5); International Institute for Population Sciences (IIPS) and ICF (6); International Institute for Population Sciences (IIPS) and ICF (31).

The World Health Organization (WHO) recommends optimal Infant and Young Child Feeding (IYCF) practices to ensure adequate child growth and development. These recommendations include initiating breastfeeding within 1 h of birth, exclusive breastfeeding for the first 6 months and continuing for 2 years or more, and providing nutritionally adequate, age-appropriate complementary feeding after 6 months (9–11). Although the IYCF practices in India have improved, they remain below optimal standards. In Assam, only 49.1% of newborns are breastfed in a timely manner and 63.8% are exclusively breastfed for the first 6 months (5).

Optimal breastfeeding could reduce under-five mortality rates by 13–19%, with an additional 6% reduction from complementary feeding (9, 12, 13). Breastfeeding also offers benefits to mothers by lowering health risks and delaying the return of fertility (14). However, women in rural areas are often found lacking appropriate knowledge and compliance with IYCF practices, which places children at risk for adverse health outcomes (15–17). Cultural and traditional feeding practices, including poor attitudes and unhealthy IYCF practices by primary caregivers, also hinder child development (18, 19).

To address these challenges, the National Institution for Transforming India (NITI) Aayog launched the ‘Aspirational Districts Transformation (ADT)’ program in 2018, targeting 115 underperforming districts across 24 states (20). The program focuses on health, nutrition, education, and various other sectors to track and improve outcomes. The Piramal Foundation also supported its implementation in 25 aspirational districts (including 5 in Assam) to enhance health and nutrition indicators.

### 1.1 Objectives

In our study, we assessed the following: (i) the knowledge, attitude, and practices (KAP) of mothers (of children aged 0–60 months) regarding recommended IYCF practices and their association with demographic characteristics and children’s nutritional outcomes; (ii) prevalence of stunting, wasting, and underweight among 0–60-months-old children; and (iii) the KAP of frontline workers (FLWs) regarding recommended IYCF practices in five districts of Assam.

## 2 Materials and methods

### 2.1 Study settings

The study was conducted in five high-focus, aspirational districts of Assam, namely Baksa (population: 988,247; health blocks: 6); Barpeta (population: 1,772,211; health blocks: 7); Darrang (population: 996,915; health blocks: 4); Dhubri (population: 2,069,000; health blocks: 7), and Goalpara (population: 1,057,927; health blocks: 6) (21). These districts have poor nutrition indicators, with high rates of stunting (48.5% in Dhubri) and underweight prevalence (37.8% in Dhubri), compared to the state averages (stunting: 35.3%; underweight: 32.8%) and national averages (stunting: 35.5%; underweight: 32.1%) (5, 7, 8). The key nutrition indicators for all five districts are provided in Table 2, based on the NFHS-5 (2019–21) data.

**Table 2:** Key nutrition indicators of all five aspirational districts of Assam.

Indicator	Assam	Baksa	Barpeta	Darrang	Dhubri	Goalpara
Children under the age of 3 years who were breastfed within 1 h of birth	49.1%	34.9%	46.4%	51.7%	57.9%	45.7%
Children under the age of 6 months who are exclusively breastfed (%)	63.6%	60.6%	62.9%	61.1%	66.4%	*
Total children aged 6–23 months receiving an adequate diet (%)	8%	14%	5.5%	9.1%	7.4%	7.5%
Children under 5 years who are stunted (height-for-age)	35.3%	41.2%	29.8%	42%	48.5%	38.9%
Children under 5 years who are wasted (weight-for-height)	21.7%	17%	19.5%	27%	21.5%	24.3%
Children under 5 years who are severely wasted (weight-for-height)	9.1%	6.2%	7.5%	9.1%	6.7%	14.3%
Children under 5 years who are underweight (weight-for-age)	32.8%	34%	26.2%	33%	37.8%	35.4%

Sources: International Institute for Population Sciences (5).

### 2.2 Sampling method and sample size

A cross-sectional study was conducted over 3 months, from April to June 2019. A multi-stage sampling technique was employed for the selection of the participants. In the first stage, a total of 10 blocks, 2 blocks from each of the 5 districts, were randomly selected to ensure adequate representation of the districts. In the second stage, 10 villages per block that conduct regular Village Health Sanitation and Nutrition Days (VHSNDs) were randomly selected. In the third stage, the sample population attending the VHSNDs and fulfilling the inclusion criteria was selected. The sample size of mothers and children was calculated using *Open Epi version 3.01*. Among the five districts, Dhubri recorded the highest prevalence of children under 3 years of age who were breastfed within 1 h of birth (57.9%) (8). Considering this prevalence, a design effect of 1.5, a precision of 5%, and a non-response rate of 10%, we calculated a sample of 398 mothers to obtain statistically significant results. Similarly, we estimated a sample size of 440 for children aged 0–60 months using a 26.2% prevalence of underweight from Barpeta (7). A total of 94 VHSNDs across the 10 health blocks were visited to collect data from 389 mothers, 456 children, and 138 frontline workers.

### 2.3 Study participants

*Inclusion criteria:* All women residing in the aforementioned five aspirational districts of Assam, primigravida and multigravida women, and mothers with children aged 0–60 months, who attended the VHSNDs in the selected villages. It also included female FLWs who were involved in community health, such as Accredited Social Health Activists (ASHA) or Anganwadi workers, who conducted the VHSNDs and consented to participate in the study. *Exclusion criteria:* Mothers or FLWs who refused to participate in the study were excluded, even if they satisfied the inclusion criteria.

### 2.4 Study variables

*Outcome variables:* The primary outcome variable (s) were the knowledge, attitude, and practices (KAP) of the mothers regarding infants and young child feeding practices. The interview schedule included 43 questions to assess the knowledge (13), attitude (10), and practices (20), based on the WHO infant and child feeding guidelines (11). If a person answered all questions correctly, they were awarded 43 scoring points. The total KAP score was categorized as good (correct answers ≥60%), average (correct answers between 40 and 59%), and poor (correct answers <40%) (22). The knowledge questions were scored with one point for correct answers and zero for wrong or uncertain responses, with a score range from 13 to 0. Individuals with more than seven correct answers (≥60%) were considered to have good knowledge of IYCF, while those with seven or fewer correct answers were considered to have poor knowledge. Attitude was assessed using a Likert scale ranging from −2 (strongly disagree) to +2 (strongly agree), with a total score ranging from a maximum of +20 to a minimum of −20. An attitude score greater than 11 (≥60%) was considered good, while a score of 11 or less was considered poor. For practices, the score range was from a maximum of +20 to a minimum of 0, with one point awarded for correct responses and zero for wrong or uncertain responses. Practices were classified as good with a score greater than 11 and as poor with a score of 11 or less. Anthropometric measurements were recorded to assess nutritional outcomes among the children aged 0–60 months, including (i) wasting (weight-for-height below −2 SD), (ii) stunting (height-for-age below −2 SD), and (iii) underweight (weight-for-age below −2 SD).

*Predictor variables:* Predictor variables were selected based on our previous knowledge of the factors affecting the knowledge, attitude and practices of the mothers regarding IYCF practices, such as sociodemographic variables (family size, socioeconomic status, religion, etc.) and environmental conditions (sanitation, drinking water, etc.).

### 2.5 Data collection

Data collection was conducted using an interviewer-administered, structured, and semi-structured interview schedule, along with an existing validated questionnaire on Infant Young and Child Feeding practices by the World Health Organization. This questionnaire was translated into the native language of the respondents to gather relevant sociodemographic characteristics, as well as the knowledge, attitude and practices of the mothers regarding IYCF. Trained staff collected anthropometric measurements of the children’s weight (kg), height (cm), and mid-upper arm circumference (MUAC) (cm). The interview schedule was pretested among 15 respondents before administering it for the data collection. These samples were not included in the main data analysis. The data obtained from the participants were kept confidential and anonymous.

### 2.6 Data analysis

The data analysis was conducted using licensed IBM SPSS statistics version 25 (23). All categorical variables were presented as frequencies and percentages, and all continuous variables were presented as mean ± standard deviation. The association between the demographic features and the mean knowledge, attitude, and practices scores was analyzed using one-way ANOVA to determine the level of significance. A *p*-value of less than 0.05 was considered statistically significant. The internal consistency of the mothers’ responses to the questions on knowledge, attitude, and practices regarding IYCF practices was assessed using Cronbach’s alpha reliability coefficient. A reliability coefficient of 0.70 or higher was considered “acceptable.” The association between stunting (below −2 SD), wasting (below −2 SD), and underweight (below −2 SD) and the mother’s KAP was assessed using the odds ratio (24, 25).

### 2.7 Ethical approval

The Institutional Ethics Committee of Piramal Swasthya Management and Research Institute (PSMRI) approved the study (IEC Study Ref No: PSMRI/2019/12). The purpose of the study was explained to participants and the Participant Information Sheet was provided in the native language of the respondent (Assamese for the study). The signed informed consent was obtained before enrolling the participants into the study.

## 3 Results

A total of 389 mothers with children aged 0–60 months participated in the study. The mean age of the mothers was 25.5 (± 5.1) years. The majority (70%) of the mothers were from the Muslim community, and more than half of them (54%) had completed schooling up to high school (class 10). The mean family size was 5.8 members (± 3.5), with an average monthly earning of INR 11,591 (± 6,611). Only one-fifth (22%) lived in pucca houses, and almost all households (97%) had access to a toilet, either kaccha or sanitary. The sociodemographic characteristics of the mothers are provided in Table 3.

**Table 3:** Comparison of the sociodemographic characteristics of the mothers with the mean KAP scores.

Characteristics	*N* = 389	Mean knowledge	*p*-Value	Mean attitude	*p*-value	Mean practice	*p*-value
District	389	7.2 (3.4)	0.00	7.4 (4.5)	0.00	10.9 (3.1)	0.00
Age (years)							
Mean (SD)	25.5 (5.1)						
16–20	72	7.6 (3.4)	0.02	9.0 (4.3)	0.00	11.3 (3.0)	0.20
21–25	132	7.6 (3.2)		7.8 (4.3)		11.0 (2.9)	
26–30	134	7.2 (3.2)		6.8 (4.2)		11.0 (3.0)	
30 and above	51	6.0 (4.0)		6.1 (5.3)		10.1 (4.1)	
Religion			0.01		0.64		0.00
Hindu	112 (29)	7.9 (3.1)		7.6 (4.5)		11.7 (2.7)	
Muslim	274 (70)	6.9 (3.4)		7.4 (4.5)		10.6 (3.3)	
Christian	3 (1)	10.3 (1.5)		9.6 (2.5)		12.3 (2.0)	
Community			0.00		0.00		0.00
General							
Socially disadvantaged	184 (62)	7.6 (2.8)		8.65 (3.9)		11.4 (2.5)	
Did not answer	111 (38)	6.0 (4.2)		4.44 (4.1)	0	9.6 (3.8)	
Language			0.00		0.00		0.00
Assamese	241 (62)	8.2 (2.9)		8.4 (4.2)		11.4 (2.8)	
Bengali	105 (27)	5.7 (2.9)		6.3 (4.3)		10.5 (2.9)	
Others	43 (11)	5.6 (4.9)		5.0 (5.1)		9.2 (4.7)	
Education			0.00		0.00		0.00
No formal schooling	52 (13)	4.7 (3.3)		3.9 (4.0)		10.0 (3.1)	
Primary	66 (17)	6.4 (2.7)		6.8 (3.2)		11.0 (2.3)	
High school (class 10)	209 (54)	7.5 (3.4)		7.9 (4.4)		10.8 (3.3)	
Higher Secondary and above	62 (16)	9.4 (2.3)		9.5 (4.7)		12.0 (3.0)	
Marital status			0.00		0.00		0.47
Married	386 (99)	7.3 (3.4)		7.5 (4.5)		10.9 (3.2)	
Widow	3 (1)	1.6 (1.1)		0.00		9.6 (0.5)	
Type of family			0.61		0.16		0.01
Joint	223 (57)	7.2 (3.5)		7.2 (4.6)		10.6 (3.4)	
Nuclear	166 (43)	7.3 (3.2)		7.8 (4.3)		11.4 (2.7)	
Number of family members			0.11		0.04		0.2
Mean, Median, SD	5.8, 5.0, 3.5	7.5 (3.1)		7.6 (4.1)		11.2 (2.6)	
Five members	221	6.8 (3.7)		7.0 (5.0)		10.6 (3.7)	
6–10 members	153	7.8 (3.9)		10.0 (4.0)		11.2 (3.3)	
More than 10 members	15						
Source of family income			0.00		0.00		0.00
Daily Wager	164 (42)	6.5 (2.9)		6.6 (3.7)		10.6 (2.3)	
Agriculture	77 (20)	5.7 (4.1)		6.0 (5.0)		9.8 (4.4)	
Government service	25 (7)	10.1 (1.6)		7.6 (3.5)		11.4 (2.5)	
Private service	21 (5)	7.1 (3.4)		7.9 (4.2)		10.1 (2.6)	
Self-employed	102 (26)	8.9 (2.9)		9.7 (4.7)		12.4 (2.9)	
Monthly income (INR)			0.00		0.00		0.00
Mean, Median, SD	11,591, 10,000, 6,611	7.7 (3.9)		9.0 (2.9)		12.1 (2.7)	
Less than 5,000	32	6.4 (3.2)		6.8 (4.1)		10.3 (3.1)	
5,000–10,000	195	7.0 (3.1)		6.6 (4.5)		10.7 (3.1)	
10,001–15,000	89	9.6 (2.8)		9.5 (5.3)		12.3 (2.8)	
More than 15,000	73						
Type of housing			0.00		0.00		0.73
Kaccha	197 (51)	6.9 (3.1)		6.8 (3.7)		11.0 (2.6)	
Semi Pucca	105 (27)	6.8 (3.8)		7.5 (5.1)		10.7 (3.7)	
Pucca	87 (22)	8.6 (3.2)		8.7 (5.1)		11.0 (3.5)	
Type of toilet at home			0.00		0.00		0.00
Not available	12 (3)	6.9 (2.6)		6.6 (3.5)		11.0 (2.0)	
Kaccha	150 (39)	6.5 (3.1)		6.5 (3.8)		10.1 (3.1)	
Sanitary	227 (58)	7.8 (3.5)		8.1 (4.9)		11.5 (3.1)	
Source of drinking water			0.00		0.00		0.00
Hand pump	340 (87)	7.2 (3.2)		7.3 (4.3)		10.8 (3.1)	
Tube well	15 (4)	3.6 (3.7)		2.5 (4.6)		9.4 (3.1)	
Public tap	12 (3)	9.2 (3.5)		9.0 (2.7)		12.1 (3.2)	
Household water supply	22 (6)	8.9 (3.4)		11.7 (4.5)		14.1 (2.3)	
Method of safe drinking water			0.00		0.00		0.00
Directly from the source	140 (36)	6.5 (2.6)		6.9 (3.0)		10.6 (2.5)	
Boiling at home	25 (6)	4.5 (4.2)		4.5 (4.2)		8.0 (3.9)	
Water filter at home	219 (56)	8.0 (3.5)		8.0 (5.1)		11.4 (3.2)	
Others	5 (1)	7.6 (2.1)		11.2 (3.5)		14.2 (1.6)	

The Cronbach’s alpha reliability coefficient was in the range of 0.81 (knowledge), 0.84 (attitude), and 0.63 (practices). The overall reliability coefficient for the 43 questions on the mothers’ KAP regarding IYCF was 0.89. Of the 389 participants, 112 (29%) had good knowledge, attitude, and practices, whereas 162 (42%) had average KAP and the remaining 115 (29%) had poor KAP regarding IYCF practices. The mean KAP scores increased significantly with improvements in variables such as community, language, education level, source of family income, monthly income, type of toilet at home, source of drinking water, and method of safe drinking water (*p* < 0.01). A comparison of the sociodemographic characteristics of the mothers with the mean KAP scores is provided in Table 3.

Of the 456 children aged 0–60 months whose anthropometric measurements were taken, 52% were male individuals and 48% female individuals, with a mean age of 20 (±15) months, an average weight of 8.7 (±2.9) kg, and a mean length of 73 (±15) cm. Approximately less than half of the children were stunted (40%, 185/456), one-third had wasting (28%, 127/456), and approximately 43% were underweight (43%, 96/456), all below −2 standard deviations, according to the WHO standards. The nutritional status of the children (0–60 months) is provided in Table 4.

**Table 4:** Nutritional status of the children (0–60 months).

	Total	Baksa	Barpeta	Darrang	Dhubri	Goalpara
		*N* (%)	*N* (%)	*N* (%)	*N* (%)	*N* (%)
	456	85 (19)	89 (19)	91 (20)	94 (21)	97 (21)
Conventional indices						
(Below −2 SD)						
Stunting (height-for-age)	185 (40)	44 (52)	21 (24)	52 (58)	53 (57)	8 (9)
Wasting (weight-for-height)	127 (28)	19 (22)	13 (15)	30 (33)	35 (38)	33 (34)
Underweight (weight-for-age)	96 (43)	33 (39)	9 (11)	47 (52)	55 (58)	51 (53)
Severe indices						
(Below −3 SD)						
Stunting (height-for-age)	106 (23)	29 (34)	9 (11)	33 (37)	24 (26)	7 (7)
Wasting (weight-for-height)	63 (14)	12 (15)	5 (6)	12 (14)	13 (14)	24 (25)
Underweight (weight-for-age)	110 (24)	14 (17)	3 (3)	25 (27)	30 (32)	38 (39)

The odds of developing stunting, wasting, and underweight among the children aged 0–60 months increased as their mother’s knowledge, attitude and practices regarding appropriate IYCF practices declined. The odds of the children developing stunting were 2.05 (CI = 1.04–4.02) times higher when their mothers’ KAP score regarding IYCF optimal practices was less than 40%, compared to those whose mothers scored more than 60%. Similar findings were reported for wasting (OR = 2.01, 0.93–4.35) and underweight (OR = 2.93, 1.47–5.85) for the mother whose KAP score was less than 40% compared to those who scored more than 60%. The association between the children’s stunting, wasting, and underweight and the mothers’ KAP score is provided in Table 5.

**Table 5:** Association between stunting, wasting, and underweight (below −2 SD) in the children (aged 0–60 months) and the KAP score of their mothers (*N* = 259).

	Stunting (height-for-age)		Wasting (weight-for-height)		Underweight (weight-for-age)	
KAP score (of mothers)	Crude Odds Ratio (OR)	*p*-value	Crude Odds Ratio (OR)	*p*-value	Crude Odds Ratio (OR)	*p*-value
Good (≥60%)	Ref^*^.		Ref^*^.		Ref^*^.	
Average (40–59%)	1.58 (0.89–2.78)	0.11	1.65 (0.85–3.21)	0.13	1.76 (0.97–3.17)	0.06
Poor (<40%)	2.05 (1.04–4.02)	0.03	2.01 (0.93–4.35)	0.07	2.93 (1.47–5.85)	0.00

KAP score: Good (correct answers ≥60%), Average (correct answers between 40 and 59%) and Poor (correct answers <40%).

* Ref. = 1.00.

**Figure 1:**
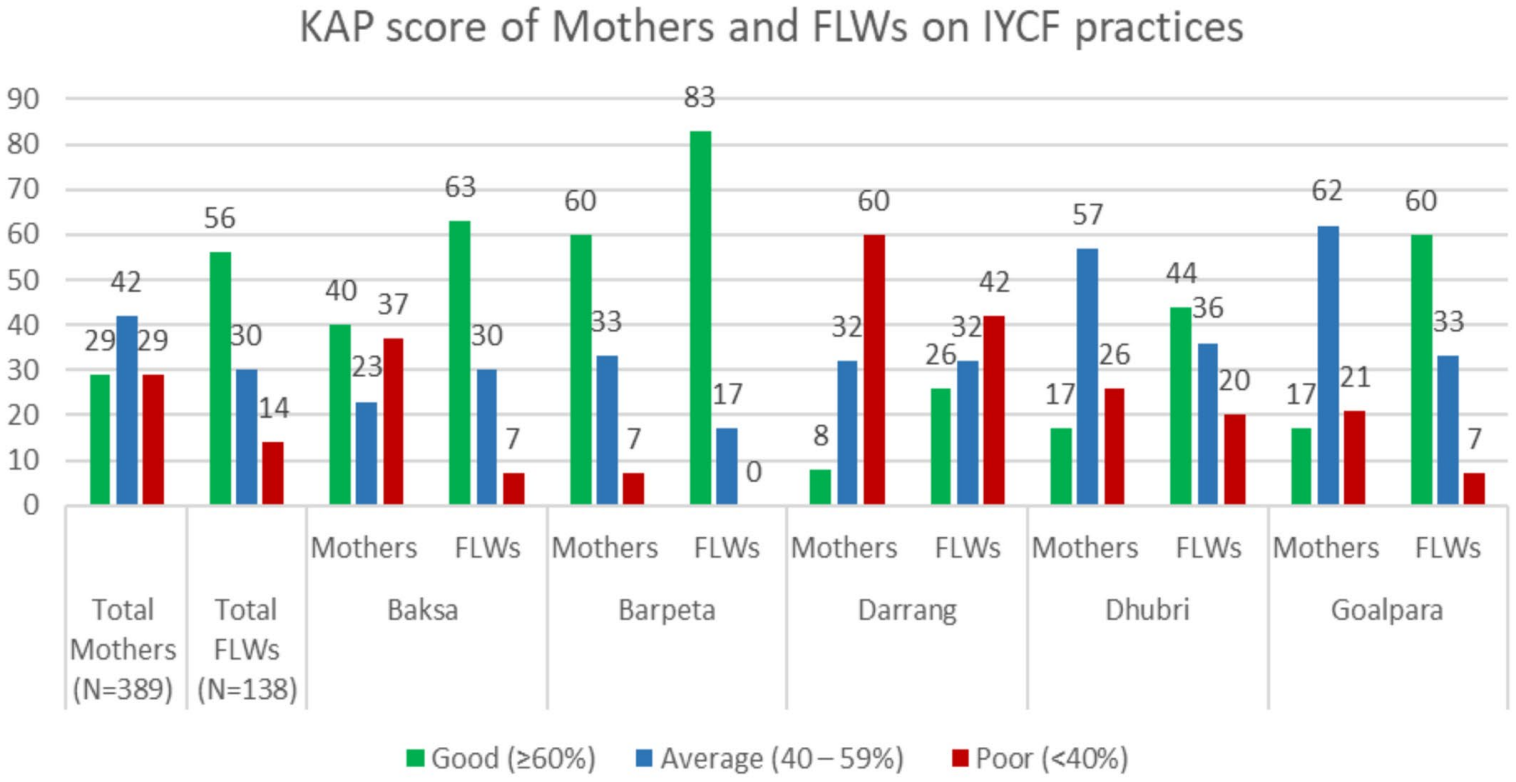
KAP scores of the mothers and FLWs regarding IYCF practices.

Of the total 138 FLWs, only 56% had good KAP scores regarding IYCF, followed by 30% with average scores and 14% with poor scores. Of the 389 mothers, 29% displayed good KAP scores, 42% had average scores, and 29% had poor scores. The KAP scores of the mothers and FLWs regarding IYCF practices are presented in Figure 1. It could be seen that the KAP of the FLWs and mothers varied widely. Despite 56% of the FLWs demonstrating good knowledge of IYCF practices, it appeared that only 29% of the mothers received this knowledge from the FLWs. Except for Baksa and Barpeta, the other three districts, where the minority population is high, showed a significant difference between the KAP of the FLWs and the mothers. Specifically, in Darrang, the difference was 26% for the FLWs and 8% for the mothers; in Dhubri, it was 44% for the FLWs and 17% for the mothers; and in Goalpara, it was 60% for the FLWs and 17% for the mothers.

## 4 Strengths and limitations

There is a lack of literature on the knowledge, attitude, and practices of mothers regarding Infant and Young Child Feeding in Assam. The strength of the study lies in the fact that we collected a sample from five different districts of Assam, which are high-priority and aspirational and have some of the poorest child nutritional indicators. There are 115 aspirational districts in India based on the composite index score (18). The findings of the present study may be applicable to other districts with similar poor health and nutrition indicators but may not be generalized to other districts of Assam.

There is a possibility of recall bias among the older mothers. The study findings will be useful for redesigning the mode of imparting programs or languages used in delivering nutrition interventions and behavior change interventions. In addition, the study provides insights into the association between a child’s nutritional outcomes and the mother’s KAP regarding IYCF practices, considering sociodemographic, maternal, and sanitation factors.

## 5 Conclusion

There is a noticeable gap in the overall knowledge, attitude and practices of the mothers of children aged 0–60 months regarding appropriate IYCF practices. These are high-priority districts in the state of Assam, with the highest levels of undernutrition, especially stunting. The present study highlights that nutritional information regarding IYCF practices is influenced by the education, language, living conditions, and economic status of the caregiver and has a positive impact on the health status of children (26–29). Despite having good knowledge, a mother may have a poor attitude toward following standard IYCF practices.

*Suggestions:* There is an urgent need for strategic information, education, and communication (IEC) activities focusing on social and behavioral change communication (SBCC) to improve both acute and chronic outcomes of childhood nutrition. It is important to note that, although the entire family was involved in the process of child feeding, none of the members were aware of the recommended guidelines. Extensive awareness interventions for all family members, along with primary caregivers, regarding IYCF practices are essential. The SBCC program for interfaith leaders had some success in bringing about a change in the community’s acceptance of health programs (30). The same strategy can be used to educate family members alongside mothers. Teenage pregnancy is also one of the key concerns in Assam. Specific interventions targeting adolescent girls should be explored to bridge the gaps in IYCF practices. The involvement of community health workers at the peripheral level is crucial for conducting continued sensitization workshops.

## Data Availability

The original contributions presented in the study are included in the article/supplementary material, further inquiries can be directed to the corresponding author.
